# Debittering of Emblica (*Phyllanthus emblica L.*) fruit powder: Preparation and biological activity

**DOI:** 10.1016/j.fochx.2023.100853

**Published:** 2024-01-02

**Authors:** Lingyu Zhang, Liting Lin, Yunxuan Hu, Daren Wu, Zhengxiao Zhang, Chaoxiang Chen, Li Wang, Jian Li

**Affiliations:** a College of Marine Food and Biological Engineering, Jimei University, Xiamen 361021, China; b Fujian Provincial Engineering Technology Research Center of Marine Functional Food, Xiamen 361021, China; c Dazhou Xinyan (Xiamen) Biotechnology Co., Ltd, Xiamen 361021, Fujian, China

**Keywords:** Emblica, Enzymolysis, Debitterization, Biological activity, Molecular docking, Simulated digestion

## Abstract

Emblica, also known as *Phyllanthus emblica L.*, is a drug homologous food that is rich in polyphenols with various biological activities. However, its bitterness and astringency pose a significant challenge to its utilization in food products. In this study, we aimed to identify the optimal conditions for debittering Emblica. Our findings revealed that the best debittering conditions were: temperature = 50 °C, pH = 4, α-l-rhamnosidase concentration 200 U/g, and time = 5 h. High-performance liquid chromatography (HPLC) and molecular docking analysis revealed that enzymatic hydrolysis partially removed bitterness compounds. The results of antioxidant activity, xanthine oxidase, and α-glucosidase inhibitory activity assays confirmed that the Emblica fruit powder still exhibited good biological activity after enzymatic debitterization. Moreover, gastric fluids treatment might contribute to the above enhancing effect of enzymatic hydrolysates of Emblica. This study provided a theoretical basis for promoting the processing and utilization of Emblica fruit powder, as well as understanding its biological activity.

## Highlight

• α-l-Rhamnosidase treated Emblica for bitterness and astringency removal, establishing the optimal enzymatic process.
• HPLC combined with molecular explored the interaction between α-l-rhamnosidase and bitter substances in Emblcia.
• The enzymatic hydrolyzates of Emblica retain biological activity, and gastric juice treatment may enhance their biological activity.

## 1 Introduction

Emblica (*Phyllanthus emblica L.*), an edible fruit, has been used as food and medicine in Asian countries such as India, China, and Malaysia for thousands of years. Emblica is rich in polyphenols, flavonoids, and other bioactive compounds (Jantan et al., 2019, Kunchana et al., 2021, Luo et al., 2012, Wu et al., 2021, Zhang et al., 2016) with multiple biological activities, such as hepatoprotective, antioxidant (Huber et al., 2009), anti-diabetic (Akhtar et al., 2011), anti-tumor (Qiu et al., 2013), anti-gout (Sato et al., 2018). However, the bitterness and astringency of Emblica can be a significant obstacle to the utilization of food products. According to studies, the bitterness and astringency of fruits are closely related to their phenolics content. Gallic acid, pyrocatechuic acid, and gallic acid are the main contributors to their bitter taste (Huang et al., 2022). Astringency is a complex phenomenon in which polyphenols or other compounds cause a drying feeling in the oral epithelium (Oladokun et al., 2016). Despite the many benefits of phenolic compounds, the bitter and astringent taste of Emblica has decreased its market value. Many attempts have been made to address this issue. Currently, debittering methods include physical adsorption debittering (Kola et al., 2010), lye treatment (Kore & Chakraborty, 2015), as well as enzymatic hydrolysis, which is an attractive way of removing bitterness and increasing the nutritional value of raw materials (Purewal & Sandhu, 2021).

Multiple studies have shown promise in this area. The enzyme α-l-rhamnosidase, has been used for the conversion of naringin, which is the bitter ingredient in citrus or grapefruit, into rhamnose and prunin (Kumar et al., 2019). Similarly, cellulase can enzymatically break down olive bitter glycosides and transform them into hydroxytyrosol, reducing the bitterness of olives (Raffaella Briante et al., 2001), Glucoamylase can hydrolyze raw or soluble starch into β-d-glucose, making it useful in a variety of industries (Norouzian et al., 2006). How these enzymes act on removing bitterness and astringency of Emblica fruit powder? This is the exact problem we want to tackle in this study. The biological activities of enzymatic hydrolysate have also been a concern. The biological activity of functional components is closely related to their digestion and absorption in the gastrointestinal digestive system. *In vitro,* simulated digestion model is a convenient, economical, efficient, and reusable tool, which is widely used to evaluate the biological activity changes of enzymatic hydrolat lysate before and after digestion and absorption.

The aim of this study was to find an effective method to improve the taste of Emblica fruit powder, while maintaining its biological activity. The optimal enzyme and enzymatic hydrolysis conditions were determined by single factor experiments and response surface optimization experiments. The content of flavonoids and the antioxidative indicators (DPPH, ABTS, hydroxyl radical scavenging ability, and ferric reducing capacity) were used during enzymatic activity and antioxidant activity evaluation, respectively. HPLC and Molecular docking were used to explore the effects and mechanism between enzyme and bitterness compounds in Emblica. Furthermore, we measured the biological activities of the enzymatic hydrolysate before and after simulated gastrointestinal digestion *in vitro*, including antioxidant capacity, xanthine oxidase, and α-glucosidase inhibitory capacity. The results of this study provided a useful reference for developing Emblica products.

## 2 Materials and methods

### 2.1 Materials

The fresh Emblica were purchased from Zhangzhou City, Fujian Province in June 2020. Food-grade cellulase (50 U/mg) was purchased from Solarbio, α-l-rhamnosidase (500 U/1.2 mL) was provided by Fujian Key Laboratory of Food Microbiology and Enzyme Engineering glucoamylase (500 U/mg) was purchased from Cangzhou Xiasheng Enzyme Biotechnology Co. LTD (Cangzhou, China). pyrocatechuic acid (CAS: 303-38-8), gallic acid (CAS: 149-91-7), syringic acid (CAS: 530-57-4), and rutinum (CAS: 153-18-4) were purchased from Shanghai Macklin Biochemical Technology Co. Analytical grade reagents for DPPH, ABTS, total reducing power, and hydroxyl radical reducing power were purchased from Sinopharm Chemical Reagent Co., Ltd (Shanghai, China). Xanthine oxidase was purchased from Sigma Aldrich, and α-glucosidase was purchased from Shanghai Yuanye Biotechnology Co., Ltd (Shanghai, China).

### 2.2 Preparation of enzymatic hydrolysate powder from Emblica

Blanch with 0.5 % sodium bicarbonate solution for 3–5 min to remove the waxiness on the surface of the Emblica fruit. After hot perching, the fruit juice was quickly cooled below 50 °C, then denucleated and juiced. The obtained fruit juice was centrifuged at 4 °C and 11,000 r/min, filtered, precipitated, and then freeze-dried into crude fruit powder. The crude filtrate powder of Emblica was dissolved in ultrapure water. After enzyme hydrolysis, the enzyme was inactivated at 100 °C for 10 min. Finally, the enzymatic powder was obtained by centrifugation, filtration, and freeze-drying.

### 2.3 Evaluation of antioxidant activity

According to the methods described previously Dong et al., 2021, Jose Carlos et al., 2020 the DPPH, ABTS and hydroxyl radical scavenging capacity were measured. DPPH powder was mixed with 60 % ethanol and configured into a 0.4 mM alcoholic solution of DPPH. The sample (100 µL) was mixed with DPPH alcohol solution (100 µL). The absorbance value (517 nm) of the mixture was determined after 30 min. The solution of 7 mM ABTS was mixed with 2.45 mM potassium persulfate solution in equal amounts and diluted with pure water until the absorbance was 0.8. The sample (50 µL) was mixed with the ABTS working solution (150 µL) and the absorbance value of the mixture (734 nm) was determined after 6 min. The sample (1 mL) was mixed with 0.2 mol/L PBS buffer solution (pH = 6.6, 2.5 mL) and 1 % potassium ferricyanide (1 mL), heated at 50 °C for 20 min, and treated with 10 % trichloroacetic acid (2 mL) and 0.1 % ferric chloride solution (1.2 mL). The absorbance value (700 nm) of the mixture was determined 20 min after the reaction was terminated. The sample (50 µL) was mixed with 9 mM ferrous sulfate solution (50 µL), 9 mM salicylic acid alcohol solution (50 µL), and 8.8 mM 30 % H_2_O_2_ solution (50 µL). The absorbance value of the mixture (510 nm) was determined after the reaction at 37 °C for 1 h. Ascorbic acid was used as a positive control.

### 2.4 Determination of total flavonoid content

The sample powder was weighed and dissolved in 60 % ethanol to prepare a sample solution of 200 µg/mL. The sample solution (960 µL) and 5 % NaNO₂ solution (640 µL) were mixed and stood for 6 min, then 10 % NaNO₂ solution (160 µL) was added and stood for 6 min, then 4 % NaOH solution (1.6 mL) was added and filled with ultrapure water to 4 mL, then stood for 15 min. The absorbance value of the mixture was determined (513 nm). The content of total flavonoids was calculated by using a rutinum gradient solution as a standard curve.

### 2.5 Selection of the optimal hydrolytic enzyme

Based on the DPPH radical scavenging ability and total flavonoid content of the enzymatic hydrolysis product, the optimal debittering enzyme was determined by comparing three candidates of cellulase, α-l-rhamnosidase, and glucoamylase.

### 2.6 Single factor and response surface optimization experiment

The study was conducted using a single factor design as follows: four factors including the amount of enzyme added and its enzymolysis time, temperature, and pH, were selected as independent variables for further study. The effects of enzymolysis time (1, 2, 3, 4, 5, 6, and 7 h), amount of enzyme added (0, 50, 100, 150, 200, and 250 U/g), temperature (20, 30, 40, 50, 60 and 70 °C) and pH (2, 3, 4, 5, 6, 7, 8, 9) on DPPH radical scavenging ability and total flavonoid content were investigated. One factor was changed while the other factors were kept constant at one time. After each hydrolysis reaction, the enzymatic solution was centrifuged, and the supernatant was taken for further analysis.

Response surface optimization experimental design used the Box-Behnken Design method (Box & Behnken, 1960). Total flavonoid content was selected as the response value, and enzymatic digestion time, temperature, and pH were selected as factors for the three-factor, three-level response surface experiment.

### 2.7 High performance liquid chromatography (HPLC)

The samples were analyzed using Agilent's 1260 Infinity II HPLC. Welch Ultimate LP-C18 (4.6 × 250 mm, 5 μm) was selected as the chromatographic column. Chromatographic conditions: HPLC was used to perform gradient elution, 0.1 % trifluoroacetic acid -Ultrapure water (A) acetonitrile (B) system as the mobile phase. The flow rate was 1 mL/min, and the injection volume was 20 μL, keep the column temperature at 35 °C and use 280 nm wavelength for detection. Elution gradient: 0 ∼ 18 min 0 %∼36 % (B) isometric elution.

### 2.8 Molecular simulation

The 3D structure of α-l-rhamnosidase (NCBI accession number AGN92963.1) and the 3D structures of all molecules (pyrocatechuic acid, CID 19; syringic acid, CID 10742; gallic acid, CID 370; rutinum, CID 5280805) were obtained from NCBI. Molecular docking was performed using VINA software, and the results were further analyzed and plotted using PYMOL. The docking algorithm was performed using the Lamarckian genetic algorithm (LGA) with the manual addition of water, the addition of polar hydrogen atoms, docking centers (−0.869, −1.151, −0.677), docking box size of (30 × 30 × 30), and a total of 30 docking times to select the best conformational docking results.

### 2.9 Sensory evaluation

The sensory evaluation panel for this study consisted of 10 individuals, including teachers and graduate students from the School of Marine Food and Bioengineering at Jimei University. The panelists’ ages ranged from 20 to 40 years old. Each panelist tasted the samples individually and rated them based on the sensory score standard outlined in Table S1 of the Taste Tasting Score.

### 2.10 Simulated digestion *in vitro*

With reference Yun et al. (2019) and with minor modifications. Simulated gastric and intestinal fluids were used to simulate digestion experiments *in vitro*. NaCl (3.1 g), KCl (1.1 g), CaCl_2_ (0.15 g), and NaHCO_3_ (0.6 g) were dissolved in ultra-pure water (1 L), then adjust pH to 1.8 with the hydrochloric acid solution, add 3000 U/mg pepsin, mix well, and configure to simulated gastric juice. The intestinal fluids were prepared as follows: NaCl (2.7 g), KCl (0.65 g), and CaCl_2_ (0.33 g) were dissolved in ultra-pure water (1 L), and the pH was adjusted to 7 with NaHCO_3_ solution, 40 U/mg trypsin was added.

Enzyme hydrolysate sample solution of 64 mg/mL and simulated gastric juice were equally mixed and digested by oscillation at 150 r/min at 37 °C. The enzymatic hydrolysis sample solution was taken at 0 h and 2 h, and the pepsin reaction was terminated at a low temperature (-20 °C, 10 min). After centrifugation and filtration, the supernatant was taken. After 2 h of simulated gastric digestion, the pH of the sample solution was adjusted to 7 and simulated intestinal solution (v/v = 1:1) was added. The mixture was digested (37 °C 150 r/min), sampled, and processed as above, and finally, the supernatant was taken.

### 2.11 Inhibition of XOD activity *in vitro*

Reference to the Ozyurek et al. (2009) method with a slight modification. Xanthine powder, p-nitro blue tetrazolium chloride (NBT) powder, and allopurinol powder were dissolved in phosphate-buffered saline (PBS buffer) to prepare 0.1 U/mL xanthine solution, 1 mM NBT solution, and 1 mg/mL allopurinol solution, respectively. Mix 25 µL sample with 75 µL PBS buffer and 25 µL XOD solution, then react for 10 min at 37 °C. Then 50 µL XOD solution and 25 µL NBT solution were added and mixed well after reacting at 37 °C for 25 min. The absorbance of the mixture was measured at 560 nm. Allopurinol solution was used as a positive control.

### 2.12 Assay of α-glucosidase inhibition

Experiments were performed according to previous study with some minor modifications (Qi & Kim, 2018). The sample (20 µL) was mixed with 0.2 U/mL α -glucoside enzyme solution (20 µL) and reacted at 37 °C for 15 min. Then 2.5 mM p-Nitrophenyl-β-d-Galactopyranoside (PNPG) solution (20 µL) was added and reacted at 37 °C for 15 min, and 1 M Na_2_CO_3_ solution (100 µL) was used to terminate the enzymatic reaction. The absorbance of the mixture was measured at 405 nm after standing for 10 min. Acarbose was used as a positive control.

### 2.13 Statistical analysis

Experimental data were analyzed by one-way analysis of variance using SPSS 25 (SPSS, Inc., Chicago, IL, USA). Data were presented as the mean ± standard deviation. Differences between groups were considered significant at *P* < 0.05 and *P* < 0.01.

## 3 Results

### 3.1 Selection of the optimal enzyme

Flavonoids were of interest because of their wide range of biological activities, but they were also controversial in the food industry because they were an important source of bitterness and astringency. Three enzymes (cellulase, α-l-rhamnosidase, and glucoamylase) were used to enzymatic hydrolysis of Emblica, and the content of total flavonoids and the DPPH IC50 were indicators of enzymolysis efficiency.

Glucoamylase-treated samples showed the lowest content of flavonoids, which was 1.56 ± 0.10 g per 100 g of Emblica enzymatic hydrolysate. For α-l-rhamnosidase and cellulose, the values were 1.87 ± 0.04 g/100 g and 1.91 ± 0.07 g/100 g, respectively. These results showed that glucoamylase had the best debittering effect, followed by α-l-rhamnosidase and cellulase. The IC_50_ of α-l-rhamnosidase hydrolysates was 34.94 µg/mL, which was lower than that of cellulase hydrolysates (IC_50_ = 40.21 μg/mL) and glucosidase hydrolysates (IC_50_ = 45.31 μg/mL). Among the three enzymes, α-l-rhamnosidase could be the optimal hydrolytic enzyme in further study. According to Adam et al. phenolic acids, flavonoids, and other polyphenolic compounds were the key active substances for both the bitterness of Emblica and its antioxidant activity (Adam & Carmen, 2000). After enzymolysis, there was a significant reduction in the total flavonoid content. Based on this observation, we hypothesized that all three enzymes could effectively eliminate the bitter compound present in the crude filtrate of Emblica (Fig. S1A). However, to fully understand the specific de-bittering effect, further analysis involving HPLC experiments and sensory evaluation is required.

### 3.2 Single factor experiments for enzymatic hydrolysis of Emblica fruit powder

#### 3.2.1 Effects of the enzyme concentration

Fig. S1B showed the amount of enzyme added to the hydrolysates of Emblica. When the amount of enzyme added was between 0 and 200 U/g, with the increment of which, the total flavonoid content in the enzymatic hydrolysates of Emblica decreased. When the amount of enzyme added was above 200 U/g, the reduction of total flavonoid was not obvious, indicating that it may have been completely hydrolysates. As for the experimental results of the antioxidant capacity analysis. When the amount of enzyme added was in the range of 0 ∼ 200 U/g, the IC_50_ DPPH increased with the increase of the enzyme concentration. When the amount of enzyme added reached 200 U/g, the IC_50_ DPPH was 34.92 μg/mL, which was close to that of the control (IC_50_ = 31.60 μg/mL), indicating that the new antioxidant active substances may be generated after α-l-rhamnosidase enzymolysis. As mentioned above, 200 U/g was selected as the enzyme addition level of α-l-rhamnosidase.

#### 3.2.2 Effects of enzymatic hydrolysis time

During the hydrolysis period, the content of total flavonoids in the hydrolysates increased first and then decreased, and finally tended to be stable **(**Fig. S1C). When the enzymolysis time was more than 4 h, the content of total flavonoids decreased slowly, indicating that prolonged time did not affect the content of total flavonoids in Emblica. The IC_50_ DPPH decreased first and then increased over time. When the enzymatic hydrolysis time was between 4 and 6 h, the IC_50_ DPPH values were lowest. Therefore, the optimal enzymatic hydrolysis time was 5 h.

#### 3.2.3 Effects of temperature on enzymatic hydrolysis

As shown in Fig. S1D, with the increase of enzymolysis temperature, the content of total flavonoids in the enzyme hydrolysate of Emblica first decreased and then increased. When the temperature of enzymatic hydrolysis was between 40 and 60 °C, the content of total flavonoids was lowest, and the antioxidant capacity also increased firstly and then decreased. Within a certain temperature range, enzymatic hydrolysis efficiency increased with the increase in temperature. When the temperature exceeds 60 °C, high temperature would lead to the decrease in the enzymatic reaction efficiency. Likewise, thermally unstable active substances were also destroyed by high temperatures, resulting in decreasing in antioxidant capacity. Above all, the optimum enzymolysis temperature range was 40 ∼ 60 °C.

#### 3.2.4 Effects of pH on enzymatic hydrolysis

As shown in Fig. S1E, between pH = 3 and pH = 7, the content of total flavonoids decreased firstly and then increased. The results showed that pH could affect the binding of the enzyme to the hydrolysates of Emblica by changing the activity and structure of the enzyme. Through analyzing the experimental results of antioxidant capacity, when the pH was at 3–5, the scavenging ability of the DPPH free radical was strongest. When pH > 7.0, the antioxidant capacity decreased sharply. Combined with the above results, it could be seen that the enzyme activity of α-l-rhamnosidase decreases under neutral and alkaline conditions, but which was better under acidic conditions. Finally, the optimal pH range of enzymatic hydrolysis was within 3–5.

### 3.3 Optimization of enzymatic hydrolysis parameters by response surface methodology

The basis of single-factor experimental results, the enzymatic hydrolysis of Emblica powder was optimized by response surface methodology (RSM). Box-Behnken Design method was used for response surface optimization experimental design. Total flavonoid content was selected as the response value, and enzymolysis time (A), enzymolysis temperature (B), and enzymolysis pH (C) were selected as factors for three-factor and three-level response surface experiments (Table S2). The Design and results of the response surface were shown in Table S3. According to Fig. S2 and Table S4, the optimal hydrolysis conditions were confirmed as follows: hydrolysis time 4.99 h, temperature 53.63 °C, pH = 4.21. Under these conditions, the predicted total flavonoid content was 1.83 g/100 g. The total flavonoid content obtained after actual enzymatic hydrolysis is 1.84 g/100 g, which was close to the theoretical value, and proved the feasibility of response surface model optimization method. Therefore, the optimal enzymatic hydrolysis conditions were determined as follows: enzymatic hydrolysis time 5 h, enzymatic hydrolysis temperature 50 °C, enzymatic hydrolysis pH = 4.

### 3.4 Analysis of HPLC results

**Fig. 1:**
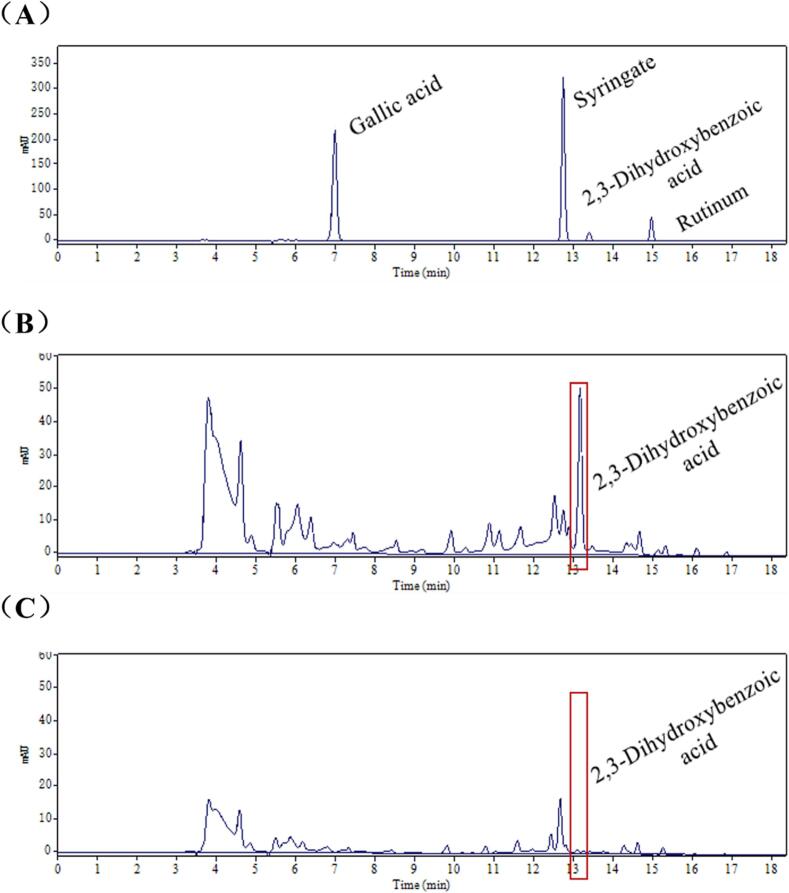
HPLC chromatogram. Mixed standards for the four bitter substances (A), the comparative illustration before and after enzymatic hydrolysis of Emblica (B)-(C).

The contents of bitter substances in Emblica before and after the treatment of α-l-rhamnosidase were determined by HPLC. The four phenolic substances were well separated under the established HPLC condition (Fig. 1A), and this method can be utilized to accurately measure these compounds contents in the samples. As shown in Fig. 1B, the peak area of gallic acid (retention time 7 min) was reduced after enzymatic digestion. The chromatographic peak of pyrocatechuic acid appeared at 13.2 min for lyophilized powder of Emblica crude filtrate. After α-l-rhamnosidase enzymolysis, the peak area at 13.2 min was significantly reduced, which showed that after α-l-rhamnosidase treatment, the bitter substance pyrocatechuic acid significantly reduced in Emblica. These results confirmed the molecular docking results above, and we indicated that α-l-rhamnosidase could reduce the content of small molecules in Emblica and could be a key enzyme for removing the bitterness of Emblica.

### 3.5 Molecular docking and visual analysis

The HPLC analysis showed that the levels of four bitter substances (pyrocatechuic acid, gallic acid, syringic acid, rutinum) in Emblica were reduced after enzymatic digestion by α-l-rhamnosidase, These results were consistent with those reported by Rojas et al. (2011), which demonstrated that the α-l-rhamnosidase could hydrolyze natural flavonoids present in fruit juices and red wines, thereby reducing bitterness and enhancing the flavor. To investigate the potential interaction mechanism between α-l-rhamnosidase and bitter substances, we performed molecular docking using VINA. The ligand–protein docking was performed 30 times, and the binding energy was calculated and compared. The best conformation was selected based on the results of Table 1. The binding energy values obtained were as follows: −7.2 Kcal/mol for pyrocatechuic acid, −6.9 Kcal/mol for gallic acid, −5.6 Kcal/mol for syringic acid when interacting with α-l-rhamnosidase. However, the binding energy of rutinum was greater than 0 Kcal/mol, indicating that rutinum and α-l-rhamnosidase may not be able to dock in their natural state. Despite the relatively good docking results for syringic acid (retention time 12.9 min), we found that it did not appear to be enzymatically hydrolyzed based on the HPLC analysis.

**Table 1:** Four small molecule docking results.

Name	Structure	Molecules Weight	Binding_Energy (Kcal/mol)
Pyrocatechuic acid		154.12	−7.2
Gallic acid		170.12	−6.9
Syringic acid		198.17	−5.6
Rutinum		610.5	>0

**Fig. 2:**
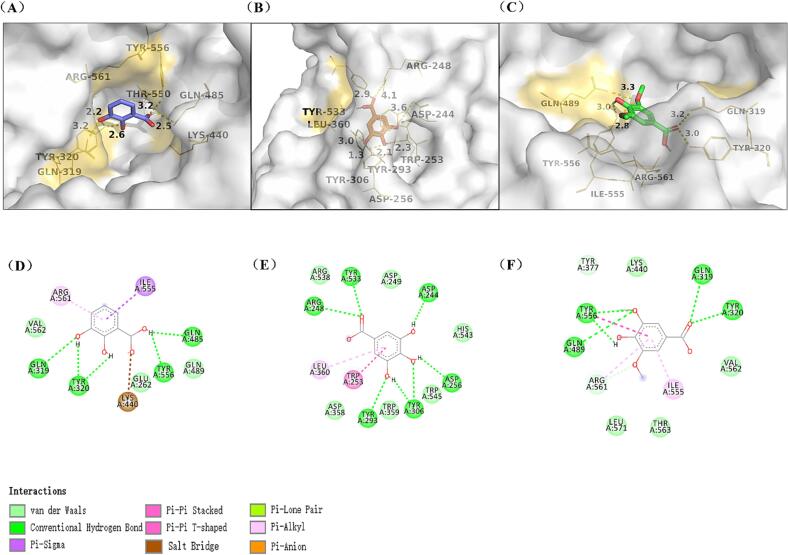
Binding patterns of α-l-rhamnosidase with the active sites of pyrocatechuic acid (A), gallic acid (B), and syringic acid (C). Visualization of the interaction results of ɑ-l-rhamnosidase with pyrocatechuic acid (D), gallic acid (E), and syringic acid (F).

In Fig. 2(A-C), the three molecular (pyrocatechuic acid, gallic acid, and syringic acid) structures were visualized by the molecular docking model. In the previous study on α-l-rhamnosidase, Li. et al. have confirmed that the (α/α) 6-barrel domain was the catalytic domain of α-l-rhamnosidase (Li et al., 2020). We found these three small molecules can enter the active cavity containing (α/α) 6-barrel domain effectively and anchored in the active cavity in the same conformational posture. The two –OH on the benzene ring of pyrocatechuic acid formed three hydrogen bonds with GLN-319 and TYR-320 of α-l-rhamnosidase with bond lengths of 3.2 Å, 2.6 Å, and 2.2 Å, respectively, and the –OH on the carboxyl group formed hydrogen bonds with GLN-485 and TYR-556 with bond lengths of 2.5 Å and 3.2 Å, respectively. It was noteworthy that the carbonyl O atom on the carboxyl group formed a salt bridge with LYS-440 (Fig. 2D). The –OH directly attached to the benzene ring of gallic acid formed five hydrogen bonds with ASP-244, ASP-256, TYR-306, and TYR-293 of α-l-rhamnosidase with bond lengths of 3.6 Å, 2.3 Å, 2.1 Å, 1.3 Å, and 3.0 Å, respectively, the O atom on the carboxyl group formed two hydrogen bonds with TYR-553 and ARG-248 and the bond length was 2.9 Å and 4.1 Å, respectively (Fig. 2B). The –OH on the syringic acid forms hydrogen bond with GLN-489 with bond length of 3.0 Å, and the O atom formed hydrogen bonds with TYR-556, GLN-489, GLN-319, and TYR-320 of α-l-rhamnosidase with the bond length was of 2.8 Å, 3.3 Å, 3.2 Å, and 3.0 Å respectively (Fig. 2C). As shown in Fig. 2(D-F), within the (α/α) 6-barrel domain of α-l-rhamnosidase, different amino acid residues form π-π conjugations with three small molecules. For example, the benzene ring of pyrocatechuic acid forms π-π conjugations with ARG-561, and ILE-555.

Observing the binding modes of the three small molecules, it was found that multiple residues within the (α/α) 6-barrel domain of α-l-rhamnosidase form hydrogen bonds with the hydroxyl groups and the O atom group of the three molecules, which indicates that these three small molecules can efficiently superimpose in the (α/α) 6-barrel domain of α-l-rhamnosidase. Among the three molecules, pyrocatechuic acid showed better binding free energy. The study by Lokhande et al. presented that hydrogen bonding is a key factor in stabilizing ligand–protein interactions (Lokhande et al., 2022). Our results showed that pyrocatechic acid formed hydrogen bonds with amino acid residues GLN-485, TYR-556, GLN-319, and TYR-320 of α-l-rhamnosidase. The O atom on the carbonyl of pyrocatechuic acid also formed a salt bridge with LYS-440 of α-l-rhamnosidase, making the interaction of pyrocatechuic acid to α-l-rhamnosidase more stable. Salt bridges played a crucial role in stabilizing protein structure and regulating protein function (Choi et al., 2021). Other chemical interactions played an important role in the combination of pyrocatechuic acid to α-l-rhamnosidase, such as π-π conjugation, van der Waals force, etc. These multiple interactions promoted the stable binding of pyrocatechuic acid within the (α/α) 6-barrel domain of α-l-rhamnosidase. Al-Shabib et al. (2018) investigated the interaction between natural polyphenols and β-lactoglobulin. They employed a combination of spectroscopic techniques, molecular docking, and molecular dynamics simulations. It is important to note that the binding sites, conformations, and interactions of bittering substances with α-l-rhamnosidase discussed earlier are primarily aimed at understanding the possible conformational relationship between these substances and enzymes. As mentioned by Mura and McAnany (2014), the results obtained from molecular docking calculations should be considered as predictive and explanatory rather than conclusive. Therefore, these methods can serve as a starting point for further scientific and systematic investigations into the mechanism of action of α-l-rhamnosidase with its substrate.

### 3.6 Sensory evaluation results

According to the experimental results of HPLC and molecular modeling, we demonstrated a reduction in the content of bitter substances in Emblica and discussed the potential molecular mechanisms of this action. These findings were further supported by in the sensory evaluation. The lyophilized powder of Emblica exhibited a golden yellow powder color with a bright and shiny appearance. It displayed good solubility and no impurities were observed upon brewing. In terms of taste, it had a slightly astringent flavor, but when compared to the lyophilized powder of crude filtrate of Emblica, the bitterness and astringency were significantly reduced. Instead, it had a sweet and sour taste with a pleasant fruity flavor similar to that of fresh Emblica juice. Importantly, it had no off-flavor, and the organoleptic evaluation yielded a score of 8.05 ± 0.39. This finding consistent with a study conducted by Gous et al. (2019) on the sensory properties and consumer preferences of grapefruit beverages. Their research indicated that consumers generally preferred beverages with lower bitterness or higher sweetness. Therefore, reducing the bitterness of Emblica could potentially enhance consumer acceptance of this product.

### 3.7 Antioxidant capacity of hydrolysates

#### 3.7.1 Antioxidant capacity of enzymatic hydrolysates

**Fig. 3:**
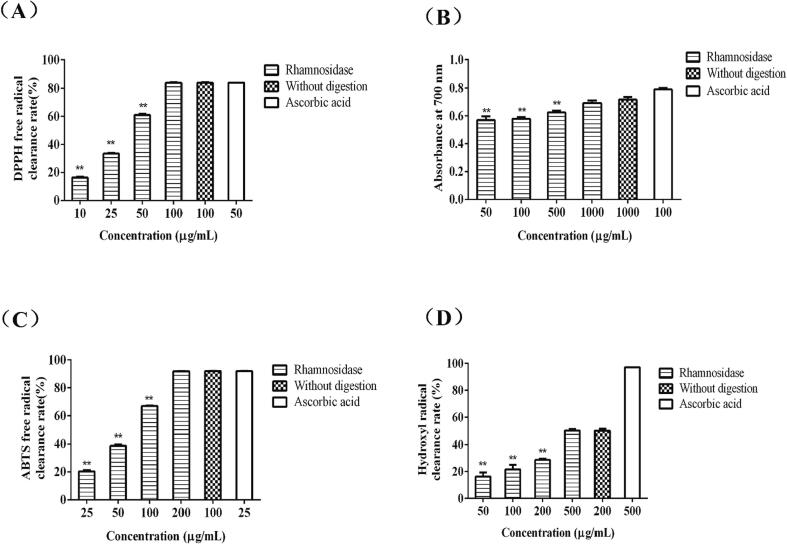
The effect of antioxidant capacity of enzymatic hydrolysates from Emblica (n = 3). DPPH radical scavenging ability (A), total reducing capacity (B), ABTS radical scavenging ability (C), hydroxyl radical scavenging ability (D). **P < 0.01 represents the comparison between the sample groups and the control group (samples without digestion).

In the experiment above, the enzymatic hydrolysis could remove the bitter substances of Emblica fruit powder, which were also important antioxidant compounds in Emblica. We further evaluated the antioxidant activities of enzymatic hydrolysates. As shown in Fig. 3A, within the range of 10–100 μg/mL, the ability of the enzymatic hydrolysates of Emblica to scavenge DPPH free radicals increased with the increase of their concentration, and the IC_50_ reached to 34.92 μg/mL. When the concentration was 100 μg/mL, there was no significant difference between the scavenging ability of enzymatic hydrolysates and the crude filtrate of Emblica at the same concentration. Total reducing power was determined by reducing Fe^3+^ to Fe^2+^, thus reflecting the total antioxidant capacity of the sample to be tested. As shown in Fig. 3B, with the increase of the concentration of the enzymatic hydrolysate of Emblica, the total reducing power gradually increased. When the enzymatic hydrolysates concentration was 1000 μg/mL, the total reducing power of the enzymatic hydrolysates was close to the crude filtrate of Emblica. Enzymatic hydrolysates solution of Emblica with different concentrations was combined with stable ABTS+ and the green solution gradually faded, thus reflecting the antioxidant capacity of the sample. As shown in Fig. 3C, within the concentration range of 25–200 μg/mL, the ABTS free radical scavenging ability of the enzyme hydrolysates of Emblica showed an increasing trend with the increase of concentration, with IC_50_ at 82.32 μg/mL. When the concentration of the enzymatic hydrolysates of Emblica was 200 μg/mL, the free radical scavenging ability of ABTS approached that of the crude filtrate at 100 μg/mL. The scavenging capacity of hydroxyl radical was related to the capacity of hydrogen proton in the sample, which could reduce the generation of hydroxyl radical and thus achieve the effect of anti-oxidation. As shown in Fig. 3D, within the concentration range of 50–500 μg/mL, the scavenging ability of hydroxyl radical of the enzymatic hydrolysates of Emblica increased with the increase of concentration. The hydroxyl radical scavenging ability of 500 μg/mL enzymatic hydrolysates of Emblica was close to that of 200 μg/mL crude filtrate. The results showed that the crude filtrate of Emblica had good antioxidant activity after enzymatic hydrolysis.

#### 3.7.2 Antioxidant capacity of digested enzymatic hydrolysates

**Fig. 4:**
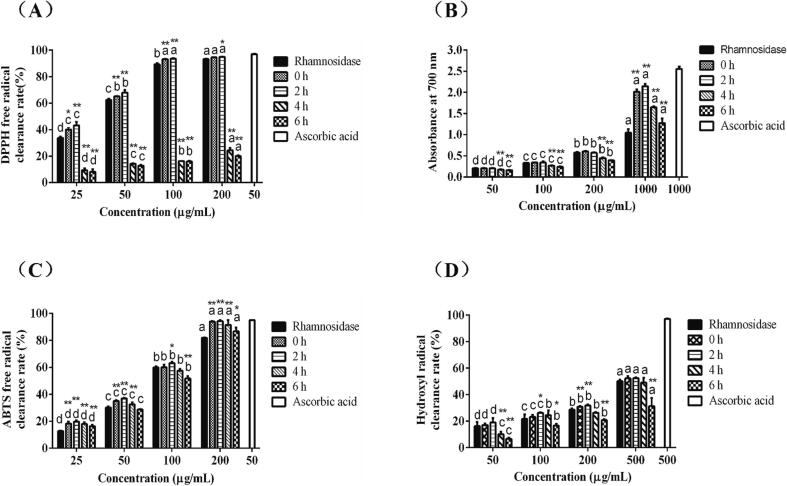
Effect on antioxidant capacity and inhibitory activity of different enzymatic digestion products (*n* = 3). DPPH radical scavenging ability (A), total reducing capacity (B), ABTS radical scavenging ability (C), and hydroxyl radical scavenging ability (D). *P < 0.05, **P < 0.01 represents the comparison between the same concentration, and the letters a to d represent the comparison of different concentrations in the same samples to be tested.

The gastrointestinal digestive environment also affected the stability of polyphenols to varying degrees, as well as their antioxidant activity. The free radical scavenging assays (DPPH, ABTS), total reducing assay, and hydroxyl free radical scavenging assay were performed to evaluate the antioxidant activity. Fig. 4(C-D) showed the antioxidant capacity of the enzyme hydrolysates of Emblica after digestion by gastric and intestinal fluids. After 2 h of exposure to gastric juice, the antioxidant ability of enzymatic hydrolysates of Emblica was increased with rising concentration. The antioxidant activity significantly decreased within 4 h after intestinal digestion. Exposure to pepsin low pH convert the bound phenols in Emblica hydrolysates into free phenols. Considering the antioxidant capacity of Emblica was related to the content of phenols and other substances, the antioxidant activity could be improved. After intestinal fluid digestion, phenolic substances were unstable and easily degraded in a neutral to slightly alkaline environment, which destroyed the antioxidant capacity of phenolic substances. It was found that the total phenolic contents of *Averrhoa carambola L.* increased after digestion with gastric juice and the antioxidant capacity increased (Mahattanatawee et al., 2006), but the total phenolic content decreased after digestion with intestinal juice, and the antioxidant capacity decreased which was consistent with the results of this study.

### 3.8 Inhibitory effect of enzymatic hydrolysates on physiological functional enzymes

#### 3.8.1 Inhibition on XOD

**Fig. 5:**
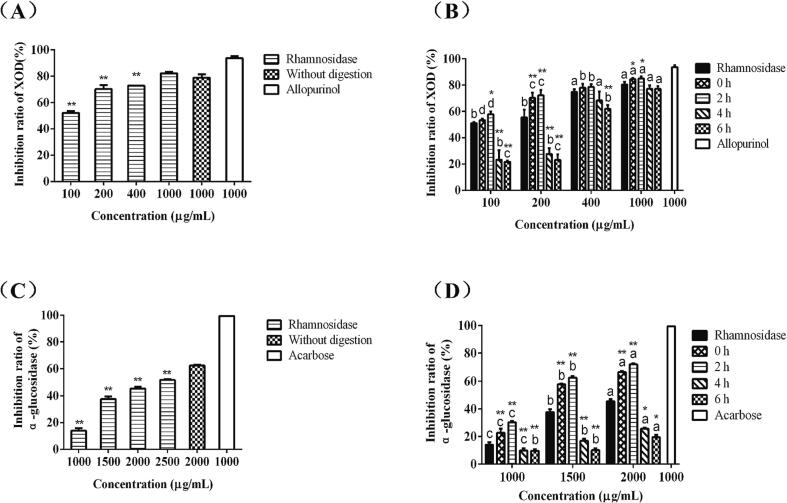
Inhibitory effect of enzymatic hydrolysates on physiological functional enzymes (*n* = 3). The effect of Emblica hydrolysates on the inhibition rate of XOD (A). **P < 0.01 represents the comparison between the sample groups and the control group (samples without digestion). The inhibitory effect of different digested products of enzymatic hydrolysates from Emblica on XOD activity (B). *P < 0.05, **P < 0.01 represents the comparison between the same concentration, and the letters a to d represent the comparison of different concentrations in the same sample groups to be tested. Effect of hydrolysates of Emblica on the inhibition of α-glucosidase (C). **P < 0.01 represents the comparison between the sample groups and the control group (samples without digestion). The effect of different digested products of enzymatic hydrolysates from Emblica on α-Glucosidase inhibitory activity (D). *P < 0.05, **P < 0.01 represents the comparison between the same concentration, and the letters a to d represent the comparison of different concentrations in the same sample groups to be tested.

XOD is a key enzyme of hyperuricemia. As shown in Fig. 5A, when the concentration of Emblica enzymatic hydrolysates ranged from 100 to 1000 μg/mL, and the inhibitory effect of the enzymatic hydrolysates on XOD increased with the increase of the dose, when the concentration of which reached 1000 μg/mL, the inhibitory effect on XOD was slightly higher than that of crude filtrate. Fig. 5B showed the inhibitory effect of the gastric juice digested products of Emblica hydrolysates on XOD. After gastric juice digestion, XOD inhibition ability of the hydrolysate samples of Emblica was significantly increased. When the concentration of digested products after intestinal juice digestion was in the range of 100–200 μg/mL, the antioxidant activity of the products was low, but it increased with the increase of the concentration. When the concentration reached 1000 μg/mL, there was no significant difference between digested and undigested Emblica hydrolyzates. The results showed that after digestion in gastric and intestinal fluid, high dose enzymic hydrolysates of Emblica had a good inhibitory effect on XOD.

Vitamin C did not have the activity of inhibiting XOD, but it could effectively reduce the activity of XOD in combination with quercetin, gallic acid, and rutinum (Zhu et al., 2004). Moreover, at pH 7.5, the strong reducing property of vitamin C protected gallic acid from being decomposed, thereby inhibiting the activity of XOD. This may help explain the results of the above experiments. Emblica contained phenolic substances such as gallic acid, as well as a large amount of vitamin C. At high doses, the phenolic substances in the enzymatic hydrolysates of Emblica were protected by vitamin C in the intestinal fluid environment, and the effect on inhibiting XOD was not significantly different from that of the blank control (samples without digestion).

#### 3.8.2 Enzymatic hydrolysates inhibition of α-glucosidase

Fig. 5C showed the inhibition of α-glucosidase by the hydrolysates of Emblica, and the inhibition was positively correlated with the concentration. When the concentration was in the range of 1000–2500 μg/mL, the inhibitory activity of Emblica hydrolysates against α-glucosidase increased with the increase of concentration, and the IC_50_ of which reached 2272.37 μg/mL, indicating that Emblica hydrolysates had inhibitory activity against α-glucosidase at high dose. Fig. 5D showed the inhibitory effects of gastric juice and intestinal juice digested products of Emblica hydrolysates on α-glucosidase. After being digested by the gastric juice, the inhibitory ability of α-glucosidase increased with the increasing concentration of enzymatic hydrolysates, which was higher than that of the control group. However, the inhibitory activity was significantly reduced after digestion in intestinal fluid.

Previous study suggested that Emblica contained active substances that inhibited α-glucosidase (Wu et al., 2022). After being digested by the gastric juice, the inhibitory effect of enzymatic hydrolysates was not significantly different from that before digestion. After being digested by the intestinal juice, the contents of total flavonoids, total phenols, and condensed tannins in the enzymatic hydrolysates were significantly reduced, and the inhibitory activity on the α-glucosidase was significantly decreased, which was consistent with the findings of our study (Zhang et al., 2018). It was speculated that the reduced inhibitory capacity of α-glucosidase was related to the reduced content of total flavonoids, total phenols, and condensed tannins, and the intestinal environment may be the main reason for the decrease of phenolic content. The enzymatic hydrolysates of Emblica contained total phenols, total flavonoids, and other substances, and the intestinal fluid environment destroyed the ability of the enzymatic hydrolysates of Emblica to inhibit α-glucosidase.

## 4 Conclusion

In this study, α-l-rhamnosidase exhibited the best effect on removing the bitterness of Emblica fruit power compared to the other two enzymes. And the optimal enzymolysis condition were temperature 50 °C, pH = 4, enzymatic digestion duration 5 h, and 200 U/g enzyme concentration. HPLC and molecular docking combined to demonstrate the removal of bitter substances. The binding of four main bitterness compounds in Emblica with α-l-rhamnosidase were investigated through molecular docking to interpret the activities of enzymes. The enzymatic hydrolysates of Emblica retained antioxidant activity and could inhibit XOD and α-glucosidase *in vitro*. Gastric fluids treatment, instead of intestinal juice, might contribute to the above enhancing effect of enzymatic hydrolysates of Emblica. The above differences might relate to the pH of the gastrointestinal fluid, which was worthy of further study. In conclusion, after being debittered by α-l-rhamnosidase, Emblica fruit powder had good antioxidant activity and had a certain potential dietary regulation effect on hyperuricemia and diabetes.

## 5 Author contributions

Conceptualization and funding acquisition, Jian Li, Li Wang; methodology, Chaoxiang Chen; project administration, Zhengxiao Zhang; data curation, Daren Wu; writing – original draft, Liting Lin, Yunxuan Hu; supervision and writing – review & editing, Lingyu Zhang.

## Declaration of competing interest

The authors declare that they have no known competing financial interests or personal relationships that could have appeared to influence the work reported in this paper.

## Data Availability

Data will be made available on request.

## References

[b0005] Adam D., Carmen G.C. (2000). Bitter taste, phytonutrients, and the consumer: A review. The American Journal of Clinical Nutrition.

[b0010] Akhtar M.S., Ramzan A., Ali A., Ahmad M. (2011). Effect of Amla fruit (Emblica officinalis Gaertn.) on blood glucose and lipid profile of normal subjects and type 2 diabetic patients. International Journal of Food Sciences and Nutrition.

[b0015] Al-Shabib N.A., Khan J.M., Malik A., Alsenaidy M.A., Rehman M.T., AlAjmi M.F., Alsenaidy A.M., Husain F.M., Khan R.H. (2018). Molecular insight into binding behavior of polyphenol (rutin) with beta lactoglobulin: Spectroscopic, molecular docking and MD simulation studies. Journal of Molecular Liquids.

[b0020] Box G.E., Behnken D.W. (1960). Some new three level designs for the study of quantitative variables. Technometrics.

[b0025] Choi M., Kim J.G., Muniyappan S., Kim H., Kim T.W., Lee Y., Lee S.J., Kim S.O., Ihee H. (2021). Effect of the abolition of intersubunit salt bridges on allosteric protein structural dynamics. Chemical Science.

[b0030] Dong R., Yu Q., Liao W., Liu S., He Z., Hu X., Chen Y., Xie J., Nie S., Xie M. (2021). Composition of bound polyphenols from carrot dietary fiber and its in vivo and in vitro antioxidant activity. Food Chemistry.

[b0035] Gous A.G.S., Almli V.L., Coetzee V., de Kock H.L. (2019). Effects of varying the color, aroma, bitter, and sweet levels of a grapefruit-like model beverage on the sensory properties and liking of the consumer. Nutrients.

[b0040] Huang H., Tan P., Li M., Tan Q., Gao J., Bao X., Fan S., Mo T., Mao W., Lin F. (2022). Quality analysis combined with mass spectrometry imaging reveal the difference between wild and cultivated Phyllanthus emblica Linn.: From chemical composition to molecular mechanism. Arabian Journal of Chemistry.

[b0045] Huber G.M., Vasantha Rupasinghe H.P., Shahidi F. (2009). Inhibition of oxidation of omega-3 polyunsaturated fatty acids and fish oil by quercetin glycosides. Food Chemistry.

[b0050] Jantan I., Haque M.A., Ilangkovan M., Arshad L. (2019). An insight into the modulatory effects and mechanisms of action of Phyllanthus species and their bioactive metabolites on the immune system. Front Pharmacol.

[b0055] Jose Carlos L.M., Leonardo S., Jesus M.C., Paola M.R., Alejandro Z.C., Juan A.V., Cristobal Noe A. (2020). Solid-state fermentation with Aspergillus niger GH1 to enhance polyphenolic content and antioxidative activity of Castilla rose (Purshia plicata). Plants-Basel.

[b0060] Kola O., Kaya C., Duran H., Altan A. (2010). Removal of limonin bitterness by treatment of ion exchange and adsorbent resins. Food Science and Biotechnology.

[b0065] Kore V.T., Chakraborty I. (2015). Efficacy of various techniques on biochemical characteristics and bitterness of pummelo juice. Journal of Food Science and Technology.

[b0070] Kumar D., Yadav S., Yadava S., Yadav K.D.S. (2019). An alkali tolerant alpha-l-rhamnosidase from Fusarium moniliforme MTCC-2088 used in de-rhamnosylation of natural glycosides. Bioorganic Chemistry.

[b0075] Kunchana K., Jarisarapurin W., Chularojmontri L., Wattanapitayakul S.K. (2021). Potential use of amla (Phyllanthus emblica L.) fruit extract to protect skin keratinocytes from inflammation and apoptosis after UVB irradiation. Antioxidants (Basel).

[b0080] Li L., Gong J., Li W., Wu Z., Jiang Z., Ni H., Li Q. (2020). Enhancement in affinity of Aspergillus niger JMU-TS528 alpha-L-rhamnosidase (r-Rha1) by semiconservative site-directed mutagenesis of (alpha/alpha)6 catalytic domain. International Journal of Biological Macromolecules.

[b0085] Lokhande K.B., Ballav S., Yadav R.S., Swamy K.V., Basu S. (2022). Probing intermolecular interactions and binding stability of kaempferol, quercetin and resveratrol derivatives with PPAR-gamma: Docking, molecular dynamics and MM/GBSA approach to reveal potent PPAR- gamma agonist against cancer. Journal of Biomolecular Structure & Dynamics.

[b0090] Luo W., Wen L., Zhao M., Yang B., Ren J., Shen G., Rao G. (2012). Structural identification of isomallotusinin and other phenolics in Phyllanthus emblica L. fruit hull. Food Chemistry.

[b0095] Mahattanatawee K., Manthey J.A., Luzio G. (2006). Total antioxidant activity and fiber content of select florida-grown tropical fruits. Journal of Agricultural and Food Chemistry.

[b0100] Mura C., McAnany C.E. (2014). An introduction to biomolecular simulations and docking. Molecular Simulation.

[b0105] Norouzian D., Akbarzadeh A., Scharer J.M., Moo Young M. (2006). Fungal glucoamylases. Biotechnology Advances.

[b0110] Oladokun O., Tarrega A., James S., Smart K., Hort J., Cook D. (2016). The impact of hop bitter acid and polyphenol profiles on the perceived bitterness of beer. Food Chemistry.

[b0115] Ozyurek M., Bektasoglu B., Guclu K., Apak R. (2009). Measurement of xanthine oxidase inhibition activity of phenolics and flavonoids with a modified cupric reducing antioxidant capacity (CUPRAC) method. Analytica Chimica Acta.

[b0120] Purewal S.S., Sandhu K.S. (2021). Debittering of citrus juice by different processing methods: A novel approach for food industry and agro-industrial sector. Scientia Horticulturae.

[b0125] Qi J., Kim S.M. (2018). α-Glucosidase inhibitory activities of lutein and zeaxanthin purified from green alga Chlorella ellipsoidea. Journal of Ocean University of China.

[b0130] Qiu Z., Zhou B., Jin L., Yu H., Liu L., Liu Y., Qin C., Xie S., Zhu F. (2013). In vitro antioxidant and antiproliferative effects of ellagic acid and its colonic metabolite, urolithins, on human bladder cancer T24 cells. Food and Chemical Toxicology.

[b0135] Raffaella Briante F.L.C., Tonziello M.P., Febbraio F., Nucci R. (2001). Antioxidant activity of the main bioactive derivatives from oleuropein hydrolysis by hyperthermophilic beta-glycosidase. Journal of Agricultural and Food Chemistry.

[b0140] Rojas N.L., Voget C.E., Hours R.A., Cavalitto S.F. (2011). Purification and characterization of a novel alkaline alpha-L-rhamnosidase produced by Acrostalagmus luteo albus. Journal of Industrial Microbiology & Biotechnology.

[b0145] Sato V.H., Sungthong B., Rinthong P.O., Nuamnaichati N., Mangmool S., Chewchida S., Sato H. (2018). Pharmacological effects of Chatuphalatika in hyperuricemia of gout. Pharmaceutical Biology.

[b0150] Wu, L., Zhang, Q., Liang, W., Ma, Y., Niu, L., & Zhang, L. (2021). Phytochemical analysis using UPLC-MS(n) combined with network pharmacology approaches to explore the biomarkers for the quality control of the anticancer tannin fraction of Phyllanthus emblica L. habitat in Nepal. *Evidance Based Complement Alternatative Medicine*. 2021, 6623791. 10.1155/2021/6623791. 10.1155/2021/6623791 PMC8018855 33833816

[b0155] Wu M., Cai J., Fang Z., Li S., Huang Z., Tang Z., Luo Q., Chen H. (2022). The composition and anti-aging activities of polyphenol extract from Phyllanthus emblica L. fruit. *Nutrients*.

[b0160] Yun L., Li D., Yang L., Zhang M. (2019). Hot water extraction and artificial simulated gastrointestinal digestion of wheat germ polysaccharide. International Journal of Biological Macromolecules.

[b0165] Zhang L., Yang S.H., Tu Z.C., Wang H., Wang T.T., Sha X.M. (2018). Influence of in vitro simulated digestion on stability and free radical scavenging and α-glucosidase inhibitory activities of Acer palmatum leaves polyphenols. Natural Product Research and Development.

[b0170] Zhang Y., Zhao L., Guo X., Li C., Li H., Lou H., Ren D. (2016). Chemical constituents from Phyllanthus emblica and the cytoprotective effects on H2O2-induced PC12 cell injuries. Archives of Pharmacal Research.

[b0175] Zhu J.X., Wang Y., Kong L.D., Yang C., Zhang X. (2004). Effects of Biota orientalis extract and its flavonoid constituents, quercetin and rutin on serum uric acid levels in oxonate-induced mice and xanthine dehydrogenase and xanthine oxidase activities in mouse liver. Journal of Ethnopharmacology.

