# Supplementary material for: Debittering of Emblica (Phyllanthus emblica L.) fruit powder: Preparation and biological activity
Source: Food Chem X. 2024 Jan 2;21:100853. doi: 10.1016/j.fochx.2023.100853 (PMC10818184; doi:10.1016/j.fochx.2023.100853)
Supplement: Supplementary data 1 [file mmc1.docx]

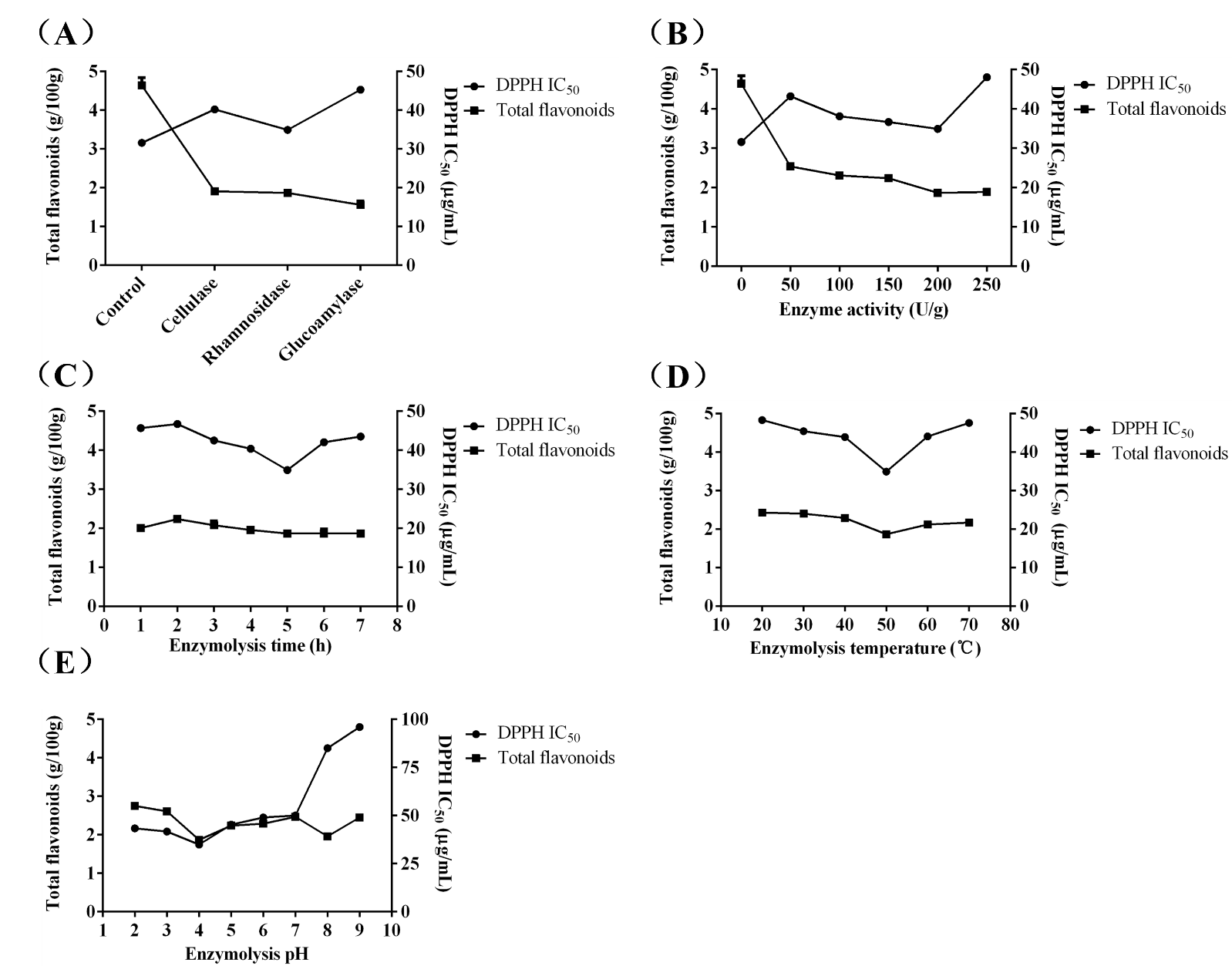


**Fig. S1** Effects of different experimental conditions on total flavonoid content (*n*=3) and antioxidation activity of enzymatic hydrolysates of Emblica. Different debitter enzymes (A), different amount of enzyme added (B), enzymatic time (C), enzymatic hydrolysis temperature (D), pH (E).


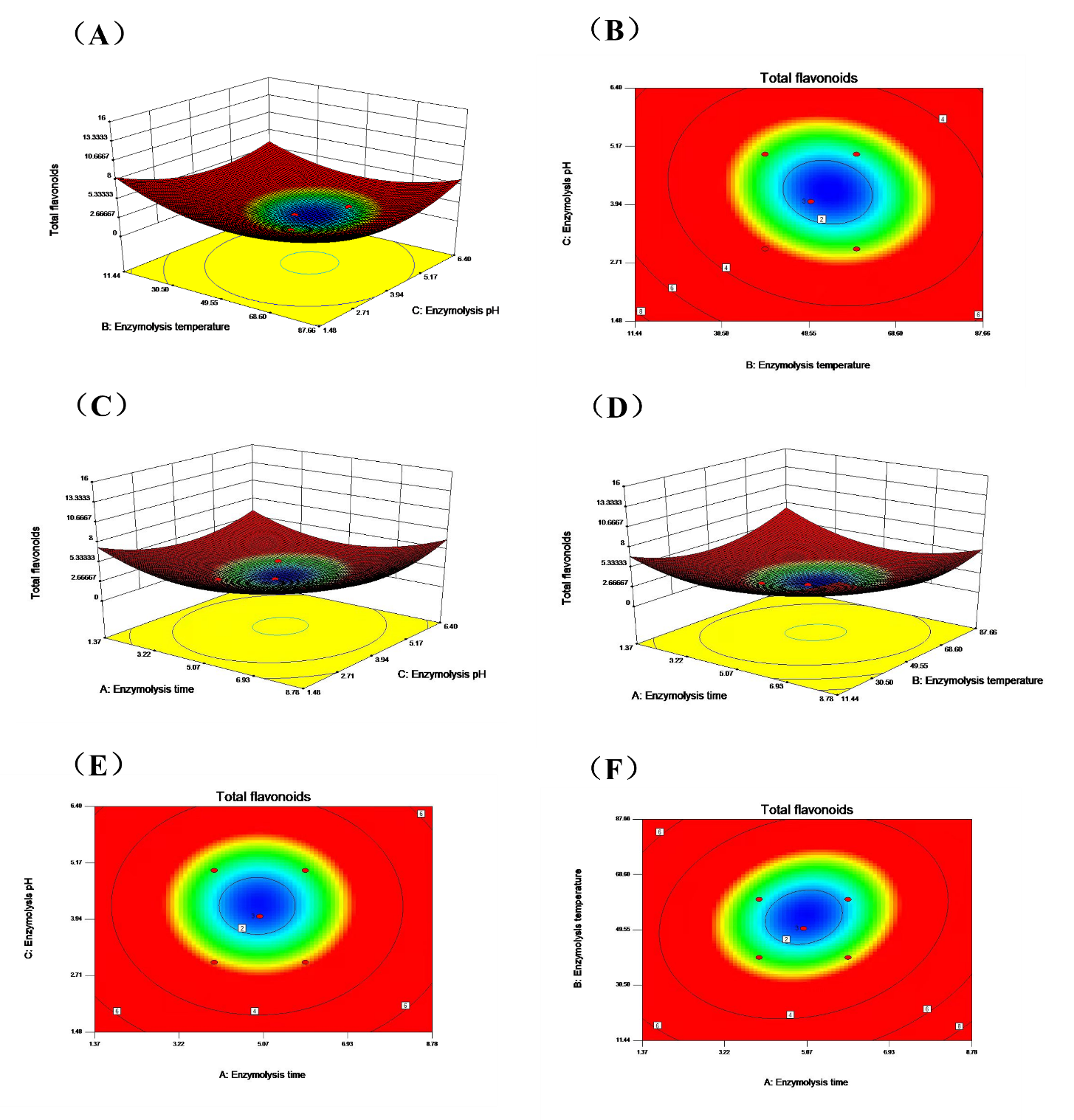


**Fig. S2** Response surface optimization results of three enzymatic hydrolysis conditions

**Table.S1**

Sensory score standard

| Scoring components | Sensory score standard | Points (10-point scale) |
| --- | --- | --- |
| Appearance and color  (20%) | The powder is dry, golden in color and shiny. | 1.5~2 |
|  | The powder is relatively dry and brown in color. | 0.8~1.4 |
|  | Powdery agglomerates, brownish color, no luster. | 0~0.7 |
| Organizational pattern  (20%) | Easy to brew and dissolve, uniform and free of impurities. | 1.5~2 |
|  | Largely soluble, with small amounts of precipitation and impurities. | 0.8~1.4 |
|  | Appearance of large amounts of precipitates and impurities. | 0~0.7 |
| Odor  (30%) | Clearly a fresh fragrance of Emblica, with no off-flavors | 2.1~3 |
|  | Smaller aroma with no off-flavor of Emblica. | 1.1~2 |
|  | Unscented or weakly scented, with off-flavors. | 0~1 |

**Table.S2**

Response surface analysis factors and levels

| Factors | Level | | |
| --- | --- | --- | --- |
|  | -1 | 0 | 1 |
| A Time（h） | 4 | 5 | 6 |
| B Temperature（℃） | 40 | 50 | 60 |
| C pH | 3 | 4 | 5 |

**Table.S3**

Response surface test design and results

| Test number | Time（h） | Temperature（℃） | pH | Total flavonoid content（g/100 g） |
| --- | --- | --- | --- | --- |
| 1 | 4 | 40 | 4 | 2.32 |
| 2 | 6 | 40 | 4 | 2.52 |
| 3 | 4 | 60 | 4 | 2.13 |
| 4 | 6 | 60 | 4 | 2.08 |
| 5 | 4 | 50 | 3 | 2.63 |
| 6 | 6 | 50 | 3 | 2.65 |
| 7 | 4 | 50 | 5 | 2.27 |
| 8 | 6 | 50 | 5 | 2.30 |
| 9 | 5 | 40 | 3 | 2.79 |
| 10 | 5 | 60 | 3 | 2.42 |
| 11 | 5 | 40 | 5 | 2.33 |
| 12 | 5 | 60 | 5 | 2.19 |
| 13 | 5 | 50 | 4 | 1.91 |
| 14 | 5 | 50 | 4 | 1.87 |
| 15 | 5 | 50 | 4 | 1.84 |

**Table.S4**

Comparison results of total flavonoids content between theoretical enzymatic hydrolysis conditions and actual enzymatic hydrolysis conditions on response surface

|  | Time  （h） | Temperature  （℃） | pH | Total flavonoid content  （g/100 g） |
| --- | --- | --- | --- | --- |
| Theoretical value | 4.99 | 53.63 | 4.21 | 1.83 |
| Actual value | 5 | 50 | 4 | 1.84 |
